# Effect of transdermal drug delivery therapy on anxiety symptoms in schizophrenic patients

**DOI:** 10.3389/fnins.2023.1177214

**Published:** 2023-06-09

**Authors:** Cuifang Zhu, Xin-Yue Wang, Jing Zhao, Bin Long, Xudong Xiao, Ling-Yi Pan, Ti-Fei Yuan, Jian-Hua Chen

**Affiliations:** ^1^Shanghai Mental Health Center, Shanghai Jiao Tong University School of Medicine, Shanghai, China; ^2^Shanghai Institute of Traditional Chinese Medicine for Mental Health, Shanghai, China

**Keywords:** schizophrenia, anxiety disorder, transdermal drug delivery therapy, efficacy, psychiatry

## Abstract

**Objective:**

To evaluate the efficacy and safety of transdermal drug delivery therapy for schizophrenia with anxiety symptoms.

**Methods:**

A total of 80 schizophrenic patients (34 males and 56 females) with comorbid anxiety disorders were randomly assigned to the treatment group (*n* = 40) and the control group (*n* = 40) with 6 weeks of follow-up. The patients in the treatment group received the standard antipsychotic drug treatment along with transdermal drug delivery therapy. The evaluation of the patients included the Hamilton Anxiety Scale (HAMA), Hamilton Depression Scale (HAMD-17), and treatment emergent symptom scale (TESS) at baseline, 3 weeks, and 6 weeks after transdermal drug delivery therapy. The Positive and Negative Symptom Scale (PANSS) was assessed at baseline and after 6 weeks of treatment.

**Results:**

After 3 and 6 weeks of treatment, the HAMA scale scores in the treatment group were lower than those in the control group (*p* < 0.001). However, there were no significant differences in the HAMD-17 scale scores, PANSS total scores, and subscale scores between the two groups (*p* > 0.05). Additionally, no significant differences in adverse effects were observed between the two groups during the intervention period (*p* > 0.05). After 6 weeks of penetration therapy, there was a low negative correlation between total disease duration and the change in HAMA scale score (pretreatment-posttreatment) in the treatment group.

**Conclusion:**

Combined traditional Chinese medicine directed penetration therapy can improve the anxiety symptoms of patients with schizophrenia and has a safe profile.

## Introduction

The importance of anxiety in schizophrenia has been recognized for a long time, and up to 65% of schizophrenic patients experience anxiety symptoms (Temmingh and Stein, 2015; Buonocore et al., 2017). Meta-analyzes and systematic reviews have demonstrated a considerable prevalence of social phobia, obsessive–compulsive disorder, posttraumatic stress disorder, panic attacks, and generalized anxiety disorder in schizophrenic patients (Achim et al., 2011; Braga et al., 2013); meanwhile, anxiety symptoms may be associated with depression, suicide, and cognitive impairment in patients and lead to increased consumption of healthcare resources (Temmingh and Stein, 2015). Sigmund Freud identified the emergence of psychotic symptoms as a defense against a potentially heightened state of anxiety, while Bluler similarly emphasized the role of affective disorders in the basic symptoms of schizophrenia. A growing body of research has highlighted the important role of anxiety in the development and recurrence of psychosis (Gomes et al., 2019) and emphasized that interventions for anxiety and emotional symptoms can play a role in the primary and secondary prevention of psychiatric disorders (Hall, 2017; Xi et al., 2021). A recent meta-analysis showed that in schizophrenic patients, the combination of antidepressants and antipsychotic medication was beneficial not only regarding affective symptoms but also psychotic symptoms (Helfer et al., 2016).

Clinical treatment of schizophrenia with anxiety disorders is still dominated by antidepressant medications, anxiolytics, and benzodiazepines (Emsley et al., 1999; Temmingh and Stein, 2015). Meta-analysis showed that second-generation antidepressants had a higher incidence of gastrointestinal adverse effects than placebo, including nausea/vomiting, diarrhoea, constipation, abdominal pain, indigestion, anorexia, increased appetite, and xerostomia (Oliva et al., 2021), and headache adverse reactions may occur with the antidepressant bupropion (Telang et al., 2018). An online investigation of the responses of 1,431 adult patients in 38 countries showed that 61% of patients reported the presence of adverse effects after using antidepressants, including drowsiness (63%) and sexual dysfunction (66%) (Read and Williams, 2018). Thus, the rate of adverse effects of antidepressants is much higher than we previously appreciated, severely affecting the quality of life of patients and often explaining their withdrawal from treatment. As a 5-hydroxytryptamine 1A receptor partial agonist, buspirone is also known to cause many adverse effects, including dizziness, nausea, fatigue, tremors, and insomnia (Strawn et al., 2018). Benzodiazepines are associated with the adverse effects of dizziness, fatigue, decreased concentration, and even addiction (Ashton, 1994; O'Brien, 2005). Neurostimulation techniques are an alternative to medication or adjunct to medication to enhance the therapeutic effect for psychiatric disorders (Gault et al., 2018; Hyde et al., 2022). Among them, transcranial magnetic stimulation (TMS) and theta burst stimulation (TBS) are noninvasive neurostimulation techniques, while deep brain stimulation (DBS) is an invasive treatment method in which stimulation electrodes are implanted in certain brain areas to send electrical stimulation (Ashton, 1994; O'Brien, 2005). A preliminary study found that TMS and TBS were effective for anxiety treatment (Chung et al., 2015). Even with the noninvasive TMS treatment method, due to the direct action on local regions, adverse effects such as the increased risk of epilepsy, headache, dizziness, and facial muscle twitching may occur, with an overall incidence of 16.7% (Stultz et al., 2020; Rossi et al., 2021). Epilepsy was the most serious complication, with an incidence of 0.16% (3/1815) (Muller et al., 2012). These factors also affect the patient’s compliance with treatment, and concerns about brain stimulation influence patients’ choice of treatment. Therefore, clinical treatments that can improve anxiety symptoms without significant adverse effects are urgently needed to improve the accompanying anxiety symptoms in schizophrenic patients.

The vine stem of *polygonum multiflorum* thunb has the functions of nourishing blood and calming the mind, dispelling wind, and promoting blood circulation. With the addition and subtraction of other drugs, many Chinese herbal formulas can be formed, which are boiled in water and taken orally. It can be used to treat insomnia, blood deficiency, body pain, rheumatism, and can also be boiled in water and applied to the affected area to treat skin itching (Liu et al., 2004). Studies have found that its effective components include stilbenes, anthraquinones, flavonoids, lecithin, tannic acid, and various trace elements (Lin et al., 2015), which have multiple pharmacological effects, including regulating the nervous system, antioxidant, immune modulation, lowering blood sugar, and reducing blood lipids (Lin et al., 2015). The main components of vine stem of *polygonum multiflorum* thunb are metabolized by the liver and kidney. Long-term use can cause a heavy burden on the liver and affect its detoxification function. Some patients may experience drug-induced liver damage (Dong et al., 2014). Transdermal drug delivery therapy is a method of external treatment in traditional Chinese medicine. Transdermal drug delivery therapy is an external Chinese medicine treatment method that uses body electrodes containing the volatile oil of the vine stem of *Polygonum multiflorum* Thunb as a substrate for drug penetration by selecting appropriate acupoints under the guidance of meridian doctrine. By means of skin administration, it avoids both the irritation of oral drugs to the gastrointestinal tract and the side effects of hepatic and renal injury and is painless and free of toxic side effects.

## Materials and methods

### Participation

Patients with schizophrenia were recruited from the inpatient department of Shanghai Mental Health Center from September 2021 to January 2022 and were randomly divided into treatment and control groups, with 40 cases in each group (with 36 male and 64 female patients, see Table 1). The inclusion criteria were as follows: (1) meeting the diagnostic criteria of ICD-10 schizophrenia and generalized anxiety disorder, (2) both patient groups received the second-generation antipsychotic medication, Olanzapine, at a dose of 10-25 mg, for 2 weeks and the dosage remained unchanged throughout the intervention period, (3) PANSS scale total score > 60, anxiety symptoms remained stable for more than 2 weeks after treatment, medication regimen remained unchanged during the intervention period, 14 points < HAMA ≤21 points, (4) 18–70 years old; (5) have more than 5 years of education; (6) determined to be free of severe physical or mental disasters, history of alcohol or drug abuse, or recent major psychological stress; and (7) signed a written informed consent form before participating in this study.

**Table 1:** Comparison of general characteristics.

	Treatment group	Control group	*t*/x^2^	*p* value
Gender				
Male	18	16	0.205	0.651
Female	22	24		
Marital status				
Married	25	21	1.035	0.793
Single	12	14		
Divorced	2	3		
Widowed	1	2		
Mean age	50.80 ± 10.99	48.90 ± 11.84	0.744	0.459
Education	10.73 ± 2.89	10.95 ± 2.855	−0.350	0.727
Course of disease	26.38 ± 10.272	25.65 ± 8.123	0.350	0.727

### Study design

**Figure 1:**
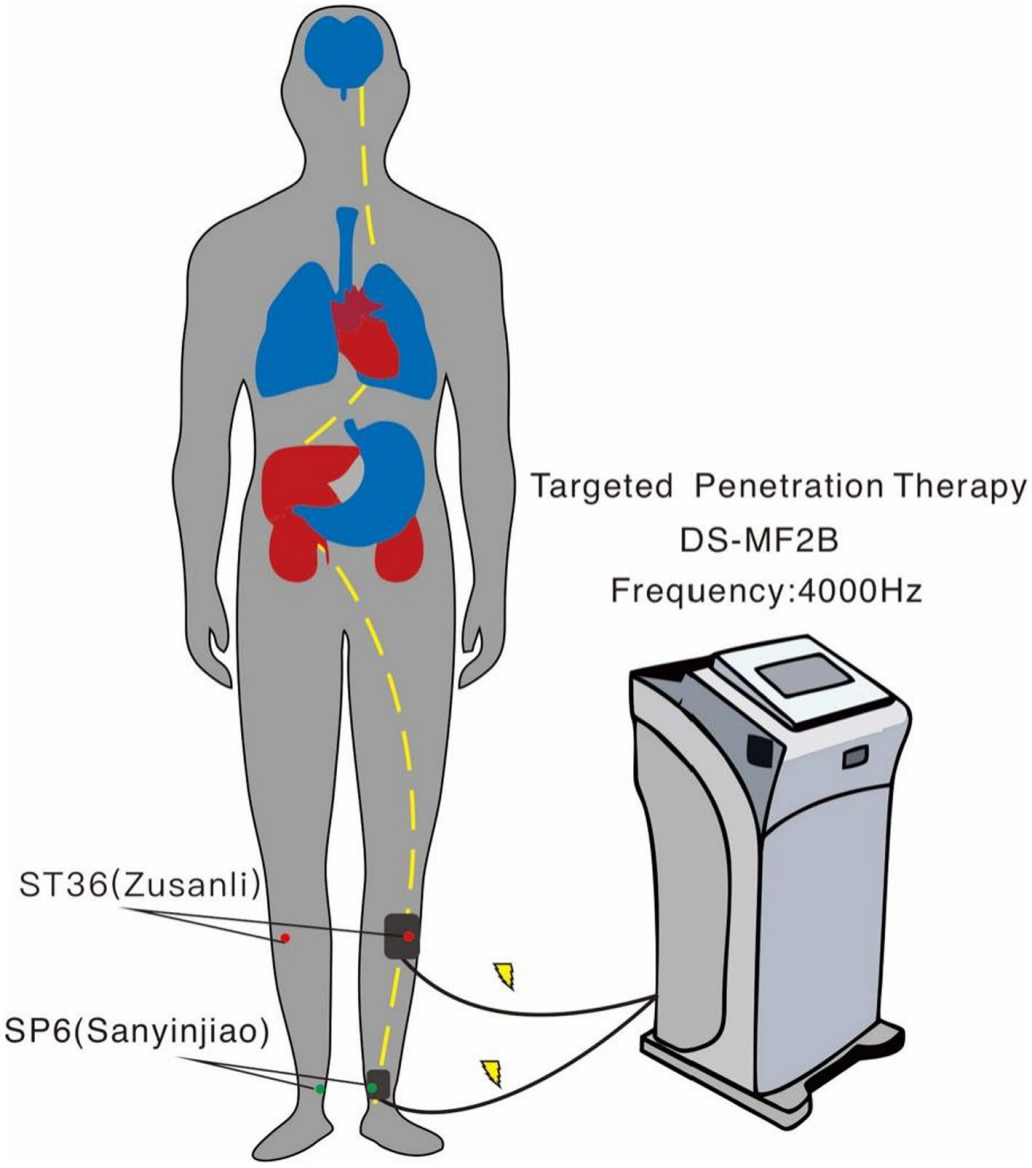
Schematic diagram of transdermal drug delivery therapy. Two acupuncture points were administered on one side of the body for 20 min; this was repeated on the other side the next day to prevent skin damage.

Based on the original second-generation antipsychotic medication and routine care, patients in the treatment group were treated with transdermal drug delivery therapy (Directional drug delivery instrument: Nanjing Ding Shi Medical Equipment Co. No. DS-MF2B, Figure 1). Each session is performed at the ST36 (Zusanli) and SP6 (Sanyinjiao) acupuncture points, and a body electrode containing volatile oil of the vine stem of *Polygonum multiflorum* Thunb is used to penetrate the herbal medicine and introduce it through the bioelectricity of the instrument. Twelve consecutive sessions were considered 1 course of treatment, with a 3-day interval between each course, for a total of 3 courses of treatment, and the entire intervention was 6 weeks. Patients in the control group were administered the original second-generation antipsychotic medication regimen and given routine care. The study was approved by the Ethics Committee of Shanghai Mental Health Center (ethical approval number: 2017-35). All patients signed an informed consent form to participate in the study.

### Treatmental index

The Hamilton Anxiety Inventory (HAMA), Hamilton Depression Inventory (HAMD-17 items), and TESS were assessed at baseline and at 3 weeks and 6 weeks of treatment. The PANSS scale consists of 7 items on the positive scale, 7 items on the negative scale, and 16 items on the general psychopathology scale to assess the severity of psychiatric symptoms. The PANSS score was assessed at baseline and 6 weeks. The TESS scale consists of 3 parts, including symptom severity, relationship with medication, and management measures, and the frequency of symptoms observed in this study. The HAMA scale was developed by Hamilton in 1959; it is one of the most commonly used scales in psychiatric practice and consists of 14 items, with all options rated on a 5-point scale from 0 to 4. The HAMD-17 was developed by Hamilton in 1960 and is the most commonly used scale to assess depressive states in clinical treatment. A total score of ≤7 is normal; a total score of 8–17 indicates mild depression; a total score of 18–24 indicates moderate depression; and a total score of ≥25 indicates major depression.

### Data statistics

SPSS 23.0 statistical software was applied for descriptive analysis and general statistics of the data. Descriptive analysis was performed for each efficacy indicator at each follow-up time point. The measurement data are expressed as the mean ± standard deviation, and the count data are expressed as percentages. The general demographic data of patients in both groups at baseline were statistically analysed by chi-square and t-tests, and the patients’ HAMA and HAMD scale scores were analysed by repeated-measures ANOVA at baseline and at weeks 3 and 6 after enrolment. The t test was used for comparison between groups, and the chi-square test was used for comparison of the incidence of adverse reactions between the two groups. Differences were considered statistically significant for values of *p* < 0.05.

## Results

### General statistics

In this study, we included 80 patients who were randomly divided into the treatment group and the control group. The treatment group was comprised of 18 male and 22 female patients, who were aged from 30 to 69 years, with 6–15 years of education and a total disease course lasting 5–49 years; additionally, 25 of the patients were married, 12 were unmarried, 2 were divorced, and 1 was widowed. The control group was comprised of 16 male and 24 female patients, who were aged 30–69 years, with 6–15 years of education and a total disease course lasting 12–39 years; additionally, 21 of the patients were married, 14 were unmarried, 3 were divorced, and 2 were widowed. No significant differences were found between the two groups in terms of age, education, total duration of illness, marital status or sex composition ratio (*p* > 0.05) (see Table 1).

### Comparison of HAMA scale scores between the two groups

At baseline, the difference between the two groups was not statistically significant (*p* > 0.05). By repeated-measures ANOVA, there was a significant difference between the group main effect (*F* = 9.270, *p* = 0.003) and a statistically significant time main effect (*F* = 61.365, *p* < 0.001) between the two groups, and there was an interaction effect (*F* = 40.957, *p* < 0.001), so simple effects analysis was used. A two-sample t-test was performed at each time point, and the HAMA scale scores of the study group were considerably lower than those of the control group after 3 weeks and 6 weeks of transdermal drug treatment (all *p* < 0.001); repeated-measures ANOVA was performed at different time points for each group with fixed grouping factors, and the statistical results showed a significant decrease in HAMA scores after treatment in the study group (all *p* < 0.05), as shown in Table 2.

**Table 2:** Comparison of HAMA scale scores ( $\bar{x}$ ±s).

	Cases	Baseline	3 weeks	6 weeks	*F* value (*p* value)			
					Group Main Effect	Time Main Effect	Interaction Effect	Simple Effects
Treatment group	40	17.80 ± 1.539	16.10 ± 1.676	15.38 ± 1.764	9.270 (0.003)	61.365 (0.000)	40.957 (0.000)	54.901 (0.000)
Control group	40	17.58 ± 1.615	17.58 ± 1.615	17.28 ± 1.797				11.323 (0.002)
t		0.638	−4.007	−4.772				
P		0.526	0.000	0.000				

### Comparison of HAMD-17 scale scores between the two groups

At baseline, the difference in HAMD-17 scale scores between the treatment and control groups was not statistically significant (*p* > 0.05). By repeated-measures ANOVA, the group main effect was not significantly different between the transdermal permeation treatment and control groups (*F* = 0.310, *p* = 0.579), and the main effect of time was significantly different (*F* = 7.583, P < =0.005), with no interaction (*F* = 1.083, *p* = 0.311), see Table 3.

**Table 3:** Comparison of HAMD-17 scale scores ( $\bar{x}$ ±s).

	Cases	Baseline	3 weeks	6 weeks	F value (*p* value)		
					Group main effect	Time main effect	Interaction effect
Treatment group	40	10.05 ± 1.037	9.93 ± 0.917	9.88 ± 0.883	0.310 (0.579)	7.583 (0.005)	1.083 (0.311)
Control group	40	10.13 ± 1.137	10.05 ± 1.061	10.05 ± 1.061			

The HAMA scale has 14 factors; items 7–13 represent somatic anxiety, while items 1–6 and 14 represent psychogenic anxiety. The change in each factor of the HAMA scale can be calculated by the change from the scores at baseline and after treatment. The changes in HAMA scores did not conform to normal distribution, so a nonparametric test (Mann–Whitney) was applied. Statistical results showed that psychogenic anxiety behaviors (tension, insomnia, behavioral manifestations during talks) and somatic anxiety behaviors (muscular system symptoms and genitourinary system symptoms) were significantly alleviated after the intervention (Z = −3.617, −5.925, −3.342, −3.087, −2.961; *p* < 0.01), see Table 4.

**Table 4:** The change in each factor of the HAMA scale [M (P25, P75)].

	Cases	Tension	Insomnia	Muscular system	Genitourinary system	Behavioral manifestations during talk
Treatment group	40	0 (0,1)	1 (0,1)	0 (0,1)	0 (0,1)	1 (0,1)
Control group	40	0 (0,0)	0 (0,0)	0 (0,0)	0 (0,0)	0 (0,0)

### Comparison of PANSS between the two groups

At baseline, no statistically significant differences were seen between the two groups in the PANSS scale total score and each subscale score (*p* > 0.05), while the comparison did not show statistically significant differences between the groups after being treated for 6 weeks (*p* > 0.05) (see Table 5).

**Table 5:** Comparison of PANSS scale total score and each subscale score ( $\bar{x}$ ±s).

Groups	Cases	Positive scale score		Negative scale score		General psychopathological scales		PANSS total score	
		Baseline	6 weeks	Baseline	6 weeks	Baseline	6 weeks	Baseline	6 weeks
Treatment	40	16.30 ± 1.757	15.93 ± 1.730	30.88 ± 2.151	30.18 ± 2.049	38.25 ± 4.174	37.98 ± 4.197	85.43 ± 5.022	84.40 ± 5.148
Control	40	16.20 ± 1.800	15.70 ± 1.522	31.48 ± 2.439	30.58 ± 2.218	39.00 ± 4.326	38.85 ± 4.246	86.68 ± 5.749	85.65 ± 5.596
t		0.251	0.617	−1.167	−0.838	−0.789	−0.927	−1.036	−1.04
P		0.802	0.539	0.247	0.405	0.432	0.357	0.304	0.302

**Figure 2:**
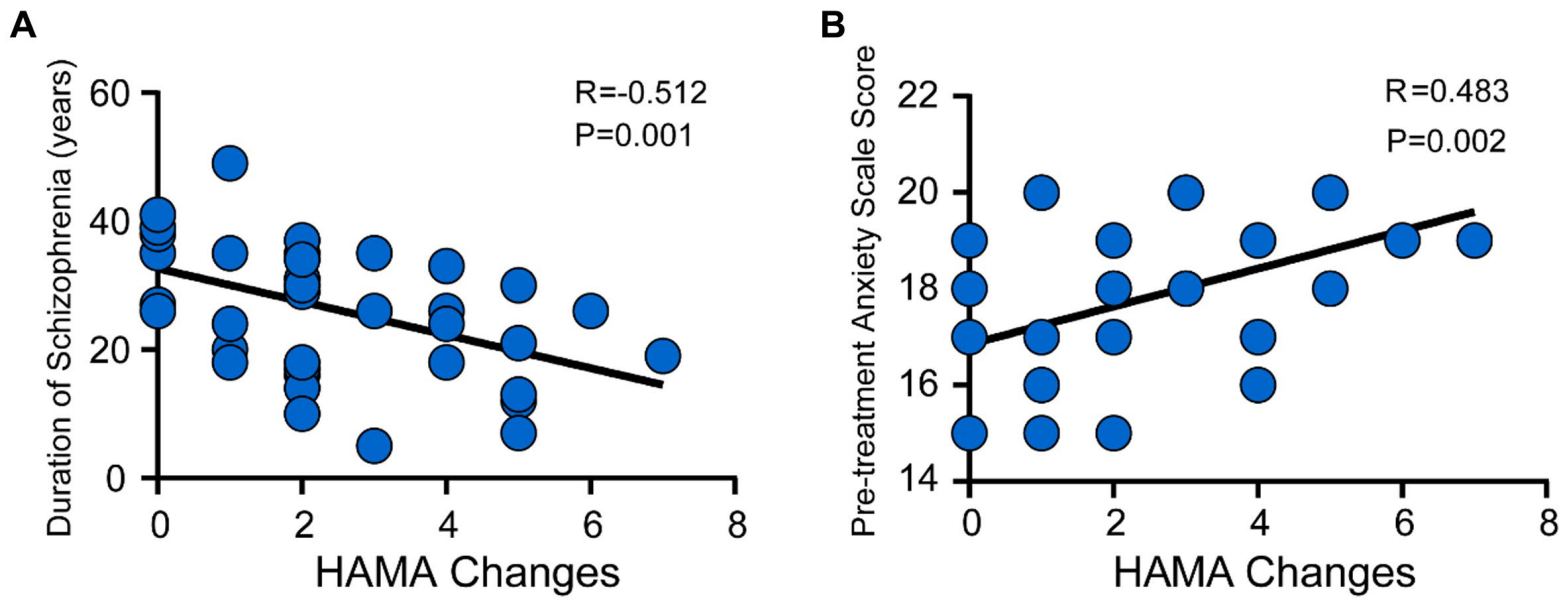
Correlation between the change in HAMA scale score with disease duration and baseline score after the intervention in the treatment group. **(A)** The correlation between total disease duration and the change in HAMA scale score (pretreatment-posttreatment) in the treatment group (R = −0.512, *p* = 0.001). **(B)** The correlation between the baseline HAMA scale score and the change in HAMA scale score (*R* = 0.483, *p* = 0.002).

We also evaluated the factors affecting the effectiveness of Chinese medicine targeted penetration therapy. As shown in Figure 2, after 6 weeks of penetration therapy, there was a low negative correlation between total disease duration and the change in HAMA scale score (pretreatment-posttreatment) in the treatment group (R = -0.512, *p* = 0.001, Figure 2A). Additionally, a low positive correlation was found between the baseline HAMA scale score and the change in HAMA scale score in the treatment group (R = 0.483, *p* = 0.002, Figure 2B).

### Comparison of side effect incidence between the two groups

There was no significant difference in the incidence of adverse effects within the TESS scale between the two groups (*p* > 0.05) (see Table 6).

**Table 6:** Comparison of the incidence of side effects.

	Cases	Hypopraxia	Insomnia	Myotonia	Tremor
Treatment group	40	2 (5.0)	3 (7.5)	2 (5.0)	3 (7.5)
Control group	40	3 (7.5)	5 (12.5)	3 (7.5)	4 (10.0)
X^2^		0.213	0.556	0.213	0.157
P		0.500	0.712	0.500	0.692

	Cases	Xerostomia	Akathisia	Diarrhoea	Dizziness
Treatment group	40	4 (10.0)	4 (10.0)	1 (2.5)	1 (2.5)
Control group	40	4 (10.0)	6 (15.0)	2 (5.0)	0 (0)
X^2^		0.00	0.457	0.346	1.013
P		1.00	0737	1.000	1.000

## Discussion

The present research investigated the efficacy of external Chinese medicine treatment methods for patients with schizophrenia with anxiety disorders. The results of this study showed that 1. At baseline, there were no significant differences in demographic and clinical parameters between the observation group and the control group (*p* > 0.05). 2. At the end of the 3rd and 6th week of intervention, patients treated with transdermal drug delivery therapy had lower HAMA scale scores compared to the control group (*p* < 0.001), indicating that transdermal drug delivery therapy can improve anxiety symptoms in patients. 3. There were no significant differences in the occurrence and frequency of adverse reactions between the two groups after intervention (*p* > 0.05), suggesting that transdermal drug delivery therapy has a high level of safety. 4. There were no significant differences between the two groups in the PANSS total score, subscale scores, and HAMD-17 scores before and after the intervention (*p* > 0.05), indicating that the 6-week transdermal drug delivery therapy had no impact on psychotic symptoms and depressive symptoms. The results of this study suggest that transdermal drug delivery therapy can improve anxiety symptoms in patients with schizophrenia and has a high level of safety.

Regarding mild anxiety disorders, psychotherapy, exercise therapy and internal and external Chinese medicine can be considered as first-line treatment to relieve anxiety while ensuring sufficient sleep and consumption of vegetables and fruits, performing more aerobic exercises, etc. For moderate and severe anxiety disorders, a combination of Chinese and Western medicinal regimens can be used to decrease drug onset time and achieve better clinical efficacy with a lower dose of drugs and fewer adverse effects. In this study, we selected Chinese medicine targeted transdermal therapy as the intervention method, which is a method of physically enhancing the delivery of topical drugs (Raphael et al., 2015) using heat therapy, bionic massage and bioelectric conduction. In the past 20 years, this technology has displayed promising performance in physiotherapy and transdermal drug delivery; for instance, its therapeutic effects have been demonstrated in dysmenorrhea, uterine cold and gynecological inflammation in obstetrics and gynecology; enlarged prostate and urinary frequency and urgency in males; adjuvant treatment in diabetes; stomach pain, diarrhoea, insomnia and other diseases. Thus, traditional Chinese targeted transdermal therapy can be used as a supplementary treatment for various ailments. In recent years, several studies have shown that Chinese medicine targeted transdermal therapy can improve the therapeutic effect of insomnia (Chen et al., 2017; Xie et al., 2019); furthermore, it has been shown that in the clinical treatment of depression, the combination of traditional Chinese targeted transdermal therapy with pharmacotherapy is highly effective and safe in improving patients’ anxiety symptoms with high efficiency and safety (Su and Huang, 2019). A study by Zhuang concluded that Chinese medicine-targeted transdermal therapy could improve anxiety in patients with somatization disorder (Zhuang, 2019). Shang et al. showed that psychological care could alleviate patients’ anxiety and improve their quality of life (Shang and Jiang, 2016). Similar results were obtained in the present study. After 3 weeks of intervention using Chinese medicine-directed transdermal therapy, the patients’ HAMA scale scores were significantly lower than those of the control group (*p* < 0.001), and no significant difference was observed in the frequency of adverse effects (*p* > 0.05), suggesting that this therapy can improve the anxiety symptoms of patients with schizophrenia and has a good safety profile. There was no significant difference between the PANSS scale total score and subscale subgroups of the two groups before and after the intervention, which suggests that Chinese medicine-directed transdermal treatment has no effect on psychiatric symptoms.

Chinese medicine classifies anxiety disorders into three types: heart deficiency and timidity, phlegm-heat disturbing the mind, and liver and kidney yin deficiency. The heart deficiency and timidity disorders may manifest as restlessness, frequent dreaming and waking up, palpitations and less food, and aversion to hearing sounds. By nourishing the heart, tranquilizing the mind, dispelling wind, and clearing ligaments, the vine stem of *Polygonum multiflorum* Thunb, as the active ingredient of transdermal drug delivery therapy can improve symptoms such as fatigue, insomnia and dreaminess and therefore can be used in the treatment of anxiety disorders. In 2020, meta-analysis researchers conducted a review of the literature on Chinese herbal medicine for insomnia with anxiety from 2000 to September 2017and concluded that the vine stem of *Polygonum multiflorum* Thunb was among the top 10 most frequently used prescribed medicines for the clinical treatment of insomnia with anxiety (Liu and Wang, 2018). For example, Danzhi Xiaoyao powder (Liu C. et al., 2021; Shi et al., 2021), which contains the vine stem of *Polygonum multiflorum* Thunb in its composition, has been shown to improve anxiety and insomnia symptoms in patients. It was also reported that extracts of the vine stem of *Polygonum multiflorum* Thunb depress the central nervous system and prolong sleep time in animals, suggesting that nightshade extracts may alleviate anxiety symptoms the next day by improving sleep quality (Sun and Wen, 2008). The inflammatory response is a major mechanism of anxiety behaviors, and studies have found that patients with anxiety disorders have significantly elevated levels of proinflammatory cytokines compared to healthy individuals (Duivis et al., 2013), meanwhile, the stem of *Polygonum multiflorum* Thunb, which has been shown to have inhibitory activities against COX-1, COX-2, and 5-LO, indicating its anti-inflammatory benefits (Li et al., 2003).

In the present study, we selected the ST36 and SP6 acupoints exert anxiolytic effects and our results demonstrate that targeted penetration therapy significantly alleviates anxiety symptoms in schizophrenic patients. ST36 (Zusanli) is a very famous acupuncture point with excellent tonic effects for treating stomach pain, indigestion, gastric distension, and hypertension and is one of Sun Simiao’s Thirteen Ghost Acupuncture Points for treating neurological disorders such as depression, anxiety, and schizophrenia (Le et al., 2016). A study found that electroacupuncture at acupoint ST36 could moderate anxiety-like behaviors in a rat model of PTSD (Liu et al., 2019). SP6 (Sanyinjiao) is the meeting point of the Spleen Meridian of ZuTaiyin, Kidney Meridian of Zushaoyin and Liver Meridian of ZuJueyin. Targeting it clinically can treat a variety of diseases, such as genital and urinary disorders (diarrhoea, bloating, dysmenorrhea, enuresis), palpitation, insomnia, hypertension, etc. A study by Du et al. (2022) indicated that electroacupuncture of SP6 could improve insomnia, anxiety, and depression symptoms in patients. The mechanisms underlying this targeted penetration therapy are currently the subject of several ongoing studies. Xing Yushuang et al. showed that acupuncture (ST36 + SP6) could alleviate anxiety in rats with alcohol withdrawal anxiety, and its anxiolytic mechanism of action was shown to be related to the regulation of cAMP-CRH interaction in hippocampal tissue in the rat brain (Xing et al., 2019). An acupuncture treatment acting on ST36 and SP6 acupoints attenuated anxiety-like behavior in rats by modulating the hippocampal inflammatory response and metabolic disorders as well as the HPA axis through effects on the gut microbiome (Zhou et al., 2022). Electroacupuncture stimulation of the ST36 acupuncture point to activate the vagal-adrenal axis to suppress systemic inflammation depends on NPY+ adrenal chromaffin cells and PROKR2^Cre^-marked sensory neurons (Liu et al., 2020; Liu S. et al., 2021). The stem of *Polygonum multiflorum* Thunb extract has anti-inflammatory activity, and stimulation of the ST36 and SP6 acupoints modulates the hippocampal inflammatory response and activates the vagus-adrenergic axis and the HPA axis; when used together, the two may jointly play an effective role in alleviating anxiety behavior in schizophrenic patients. In our subsequent studies, emphasis will be placed on the biological mechanisms of traditional Chinese medicine permeation therapy to better facilitate its application in clinical treatment.

The current study conducted a correlation analysis of the potential factors influencing the effectiveness of targeted penetration therapy, revealing that the longer the duration of schizophrenia, the worse the treatment effect. In traditional Chinese medicine theory, the initial stage of anxiety disorder is mostly characterized by mental symptoms such as nervousness and insomnia, and as the disease progresses, somatic anxiety becomes the main symptom, manifesting as the muscular system and genitourinary system symptoms, which increases the difficulty of treatment. This is also consistent with the literature reports that a longer duration of untreated psychosis and relapses are modestly related to worse outcomes, and early antipsychotic treatment in the prodromal stage can achieve better results (Sarotar et al., 2008; Fountoulakis et al., 2020). This study also found that the higher the baseline HAMA scale (i.e., the more severe the underlying anxiety symptoms), the more obvious the improvement in anxiety after traditional Chinese Medicine treatment. In conclusion, our results suggest a correlation between the improvement effect of treatment on anxiety disorders in patients with schizophrenia and the duration of illness and baseline anxiety level, providing a theoretical basis for large-scale clinical implementation. Patients with schizophrenia with a short duration of disease and significant anxiety symptoms should be targeted for treatment.

In conclusion, transdermal drug delivery therapy, as a simple and convenient method of external Chinese medicine treatment, using vine stem of *polygonum multiflorum* thunb as the penetrant and acupoints of ST36 + SP6, significantly improves the anxiety symptoms of patients with schizophrenia, with minimal adverse effects and a good safety profile. However, this study had a small sample size and was conducted as a preliminary exploratory study. There was no testing of inflammatory factors, serotonin, dopamine, or other biomarkers that may be related to anxiety before and after intervention, nor was there a breakdown of the active ingredients in the vine stem of *polygonum multiflorum* thunb. These limitations are regrettable for identifying the effective components of the treatment and the biological markers. Nevertheless, our study provides a new approach to improving anxiety in patients with schizophrenia, and future studies need to be conducted on a larger scale in multiple centers with more comprehensive research designs, including a group treated with “body electrode with bioelectricity of the instrument” as a control and further clarification of the biological mechanisms of targeted transdermal drug delivery through animal experiments.

## Data availability statement

The raw data supporting the conclusions of this article will be made available by the authors, without undue reservation.

## Ethics statement

The studies involving human participants were reviewed and approved by the Ethics Committee of Shanghai Mental Health Center (ethical approval number: 2017-35). The patients/participants provided their written informed consent to participate in this study.

## Author contributions

JZ and BL formulated the design of the studies. CZ and X-YW performed the experiments and analysis of the studies and drafted the manuscript. CZ, X-YW, XX, and L-YP performed the experiments and collected data. J-HC reanalysis the data and helped revise manuscripts and draw tables. All authors contributed to the article and approved the submitted version.

## Funding

This study was supported by Shanghai Municipal Health Commission Key Training Program of Traditional Chinese Medicine (No: AB83180002019021); Three-year Action Plan Project of Shanghai Traditional Chinese Medicine Development (grant ZY-(2021–2023)-0207-01); “Scientific and technological innovation action plan” of Shanghai Science and Technology Commission of China (grant 21Y11921100), the special project of integrated Chinese and western medicine in Shanghai General Hospital (ZHYY-ZXYJHZX-202004).

## Conflict of interest

The authors declare that the research was conducted in the absence of any commercial or financial relationships that could be construed as a potential conflict of interest.

## Publisher’s note

All claims expressed in this article are solely those of the authors and do not necessarily represent those of their affiliated organizations, or those of the publisher, the editors and the reviewers. Any product that may be evaluated in this article, or claim that may be made by its manufacturer, is not guaranteed or endorsed by the publisher.
